# Dual time point based quantification of metabolic uptake rates in ^18^F-FDG PET

**DOI:** 10.1186/2191-219X-3-16

**Published:** 2013-03-13

**Authors:** Jörg van den Hoff, Frank Hofheinz, Liane Oehme, Georg Schramm, Jens Langner, Bettina Beuthien-Baumann, Jörg Steinbach, Jörg Kotzerke

**Affiliations:** 1 PET Center, Institute of Radiopharmaceutical Cancer Research, Helmholtz-Zentrum Dresden-Rossendorf, Dresden, 01328, Germany; 2 Department of Nuclear Medicine, University Hospital Carl Gustav Carus, Technische Universität Dresden, Dresden, 01307, Germany

**Keywords:** Whole-body PET, Dual time point, Metabolic rate of FDG, PET quantification, Tracer kinetic modeling

## Abstract

**Background:**

Assessment of dual time point (DTP) positron emission tomography was carried out with the aim of a quantitative determination of *K*_*m*_, the metabolic uptake rate of [^18^F]fluorodeoxyglucose as a measure of glucose consumption.

**Methods:**

Starting from the Patlak equation, it is shown that $K_{m}\approx m_{t}/c_{a}^{0}+{\bar{V}}_{r}/\tau_{a}$, where *m*_*t*_ is the secant slope of the tissue response function between the dual time point measurements centered at *t* = *t*_0_. $c_{a}^{0}=c_{a}(t_{0})$ denotes arterial tracer concentration, ${\bar{V}}_{r}$ is an estimate of the Patlak intercept, and *τ*_*a*_ is the time constant of the *c*_*a*_(*t*) decrease. We compared the theoretical predictions with the observed relation between $K_{s}=m_{t}/c_{a}^{0}$ and *K*_*m*_ in a group of nine patients with liver metastases of colorectal cancer for which dynamic scans were available, and *K*_*m*_ was derived from conventional Patlak analysis. Twenty-two lesion regions of interest (ROIs) were evaluated. *c*_*a*_(*t*) was determined from a three-dimensional ROI in the aorta. Furthermore, the correlation between *K*_*m*_ and late standard uptake value (SUV) as well as retention index was investigated. Additionally, feasibility of the approach was demonstrated in a whole-body investigation.

**Results:**

Patlak analysis yielded a mean *V*_*r*_ of ${\bar{V}}_{r}=0.53\pm 0.08$ ml/ml. The patient averaged *τ*_*a*_ was 99 ± 23 min. Linear regression between Patlak-derived *K*_*m*_ and DTP-derived *K*_*s*_ according to *K*_*s*_ = *b* · *K*_*m*_ + *a* yielded *b* = 0.98 ± 0.05 and *a* = -0.0054 ± 0.0013 ml/min/ml (*r* = 0.98) in full accordance with the theoretical predictions *b* = 1 and $a\approx -{\bar{V}}_{r}/\tau_{a}$. *K*_*s*_ exhibits better correlation with *K*_*m*_ than late SUV and retention index, respectively. $K_{s}^{\left(c\right)}=K_{s}+{\bar{V}}_{r}/\tau_{a}$ is proposed as a quantitative estimator of *K*_*m*_ which is independent of patient weight, scan time, and scanner calibration.

**Conclusion:**

Quantification of *K*_*m*_ from dual time point measurements compatible with clinical routine is feasible. The proposed approach eliminates the issues of static SUV and conventional DTP imaging regarding influence of chosen scanning times and inter-study variability of the input function. *K*_*s*_ and $K_{s}^{\left(c\right)}$ exhibit improved stability and better correlation with the true *K*_*m*_. These properties might prove especially relevant in the context of radiation treatment planning and therapy response control.

## Background

For many years, quantification of the metabolic rate of glucose consumption with dynamic [^18^F]fluorodeoxyglucose (FDG) positron emission tomography (PET) using the so-called Patlak plot, a procedure most clearly described by Patlak in his seminal papers [1,2], has proven valuable in PET research and clinical routine.

However, in the clinical oncological setting, quantification is mostly restricted to the ubiquitously used standard uptake value (SUV). The reason is twofold: (1) no need (or even inability) to determine the arterial input function (AIF) and (2) inability to perform dynamic whole-body investigations.

Without question, the SUV (defined as the tracer uptake at a certain time point normalized to injected dose per unit body weight) has proven a valuable means of achieving a certain level of quantitative description, thus allowing, e.g., definition of standardized evaluation schemes (see [3] for an overview).

The approach, however, has known shortcomings [4-6]. SUVs do not directly provide information about the tracer kinetics but, by their very nature, only a static snapshot somewhere on the tissue response function (TRF). Naturally, SUVs are varying along the given TRF and are thus prone to variability when not determined at a strictly standardized time. Since SUVs do not contain any information of the actual rate of tracer accumulation (related to the slope of the tissue response function), TRFs from different tissues might in extreme cases even intersect at a certain time (thus exhibiting identical SUVs and zero image contrast at this moment) while having completely different kinetic properties. Tissue SUV stability is further compromised by not accounting for the sizable inter-study variability of arterial blood SUV which directly influences the actually obtained tissue uptake.

One quite extensively investigated way around the ‘snapshot problem’ is dual time point (DTP) investigations [7,8] in which two successive whole-body scans are performed to obtain information regarding the rate of tracer accumulation. While being undoubtly valuable in discriminating between tumor and inflammation, quantitative evaluation of DTP measurements is usually restricted to computation of a so-called retention index, RI, representing the percentage change of SUV_max_ or SUV_mean_ between early and late images (see, e.g., [9,10]). However, the retention index, too, depends on the acquisition time of (and time difference between) early and late PET scan and, therefore, requires the same strict standardization as the SUV approach to provide useful quantitative measures. The retention index, too, is affected by the mentioned AIF variability at late times due to the evoked changes of the TRF slope.

There also have been attempts to directly use the TRF slope obtained in dynamic scans as a substitute for actual kinetic modeling [11,12] while avoiding measurements of tracer concentration in blood. However, a convincing physiological interpretation of the slope parameter is missing. Furthermore, the approach suffers from the same problems as SUVs and retention index regarding the uncontrolled influence of the inter- and intra-subject variability of the AIF.

In this study, we propose a new assessment of DTP (and, more generally, TRF slope)-based methods with the aim of a quantitative determination of *K*_*m*_, the metabolic uptake rate of FDG. We demonstrate that starting from the Patlak model, one can derive an analytical relation between *K*_*m*_ and the TRF slope *m*_*t*_, which only requires the image-based determination of the AIF during the respective late PET scans. The derived relation is especially compatible with dual time point whole-body investigations.

In this retrospective investigation, we evaluate the new approach in a group of patients with liver metastases of colorectal cancer for which *K*_*m*_ was determined, both, by conventional Patlak analysis of the fully dynamic PET scans as well as by the newly developed approach.

## Methods

### Theory

**Figure 1:**
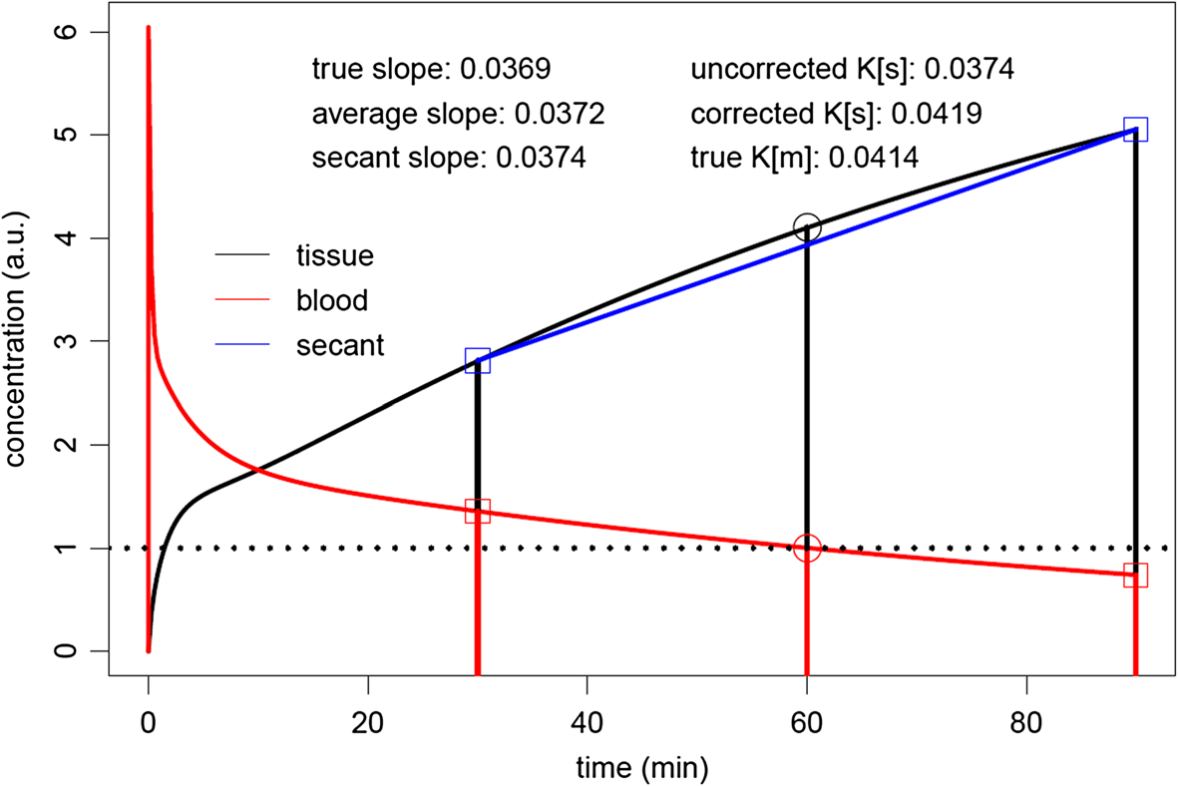
**AIF plus TRF calculated for *K***_**1 **_**= 0.3 ml/min/ml, *k***_**2 **_**= 0.5 /min, *k***_**3 **_**= 0.08 /min.** The TRF does not become linear at later times but exhibits a visible curvature. However, the slope at some time point *t*_0_ (*t*_0_ = 60 min in this example) is nearly identical to the slope of the secant connecting the boundary points of a finite time interval centered at *t*_0_. The AIF is scaled such that *c*_*a*_(*t*_0_) = 1. The data accessible in a DTP measurement are indicated by the square plotting symbols. For further details, see the main text.

It is well known that the TRF after a bolus injection of FDG appears to be approximately linear at later times. Closer inspection, however, reveals, that the curve exhibits a finite curvature: the slope decreases with time due to the continuously decreasing AIF (see Figure 1). In the Appendix, we demonstrate that for times *t* when the Patlak equation is valid (usually for *t* > 20-30 min), the ratio between the instantaneous values of TRF slope and AIF level can be expressed in terms of the parameters *K*_*m*_ and *V*_*r*_ of the Patlak model and the time constant *τ*_*a*_ describing the essentially mono-exponential decrease of the AIF in the considered time window. It is shown in the Appendix that the TRF slope at *t* = *t*_0_ is very nearly identical to the slope of the secant connecting the boundary points of a finite symmetric time interval around *t*_0_ (and also to the average slope in this interval).

One finally arrives at the relation

(1) $$K_{m}=K_{s}+\frac{V_{r}}{\tau_{a}}=\frac{m_{t}}{c_{a}^{0}}+\frac{V_{r}}{\tau_{a}}$$

with

(2) $$K_{s}=\frac{m_{t}}{c_{a}^{0}},$$

where *m*_*t*_ is the secant (or average) TRF slope in the chosen time interval centered at *t*_0_ and $c_{a}^{0}=c_{a}(t_{0})$ (see Figure 1).

The rate *K*_*s*_ defined by Equation 2 (i.e., the ratio between the TRF slope and AIF level at time *t*_0_) can be determined from measurements during the late phase alone. Contrary to the Patlak method, knowledge of the full AIF is not required. To the extent that *K*_*s*_ ≫ *V*_*r*_ / *τ*_*a*_, *K*_*s*_ might directly serve as an (negatively biased) approximation of *K*_*m*_. Moreover, to the extent that *V*_*r*_ can be replaced by a suitable constant value ${\bar{V}}_{r}$, *K*_*s*_ differs from *K*_*m*_ only by a *τ*_*a*_- dependent offset that can be added to *K*_*s*_ to obtain a corrected value

(3) $$K_{s}^{\left(c\right)}=K_{s}+K_{0}=K_{s}+\frac{{\bar{V}}_{r}}{\tau_{a}}$$

that approximates *K*_*m*_ quite accurately (see Appendix and Figure 1).

We have compared these theoretical predictions with the actually observed relation between *K*_*s*_ and *K*_*m*_ in a group of patients with liver metastases for which fully dynamic scans were performed.

### Study sample

The investigated patient group included nine male subjects with liver metastases of colorectal cancer (mean age 62.8 years, range 48 to 76). For each patient, one to three dynamic PET scans of 60 min duration were performed (altogether 15 scans). Scans started immediately after injection of 346 to 430 MBq FDG. The scans were performed with an ECAT EXACT HR ^+^ (Siemens/CTI, Knoxville, TN, USA). The acquired data were sorted into 23 to 31 frames with 10 to 20 s duration during bolus passage, 30 to 150 s duration until 10 min post-injection (p.i.), and 300 s duration afterwards. Tomographic images were reconstructed using attenuation-weighted OSEM reconstruction (6 iterations, 16 subsets, 6 mm FWHM Gaussian filter).

Additionally, feasibility of the generation of parametric $K_{s}^{\left(c\right)}$ maps was demonstrated in a whole-body FDG investigation of a 63-year-old woman with bronchial carcinoma of the right lung and lymph node metastases of the right hilar region and the mediastinum (Philips Ingenuity TF PET/MR (Philips, Cleveland, OH, USA), injected dose 273 MBq, first scan 67 min p.i. (2 min per bed position), second scan 117 min p.i. (1.5 min per bed position)).

### Data evaluation

Region of interest (ROI) definition was performed using ROVER (ABX, Radeberg, Germany) [13,14]. The AIF was determined from a roughly cylindrical three-dimensional (3D) ROI centered in the aorta using a concentric safety margin of at least 1 cm to exclude partial volume effects. 3D lesion ROIs were defined in 22 lesions, and the respective TRFs were computed. Further data analysis was performed using the R software for statistical computation [15].

For all 22 lesions, *K*_*m*_ and *V*_*r*_ were derived from the conventional Patlak analysis of the full dynamic data later than 20 min p.i. (at which time all Patlak plots already were linear). For comparison with the corresponding result of the subsequent DTP evaluation, *τ*_*a*_ was determined from a mono-exponential fit to the complete AIF data in the time window used for the Patlak analysis. Variability of *τ*_*a*_ and *V*_*r*_ was expressed as mean ± standard deviation (SD).

Dual time point data were generated from the data 20 to 30 min and 50 to 60 min p.i., yielding two pairs of *c*_*a*_ and *c*_*t*_ values which were assigned to the respective frame centers *t*^ - / + ^ = 25 / 55 min (which corresponds to *t*_0_ = 0.5 · (*t*^-^ + *t*^+^) = 40 min and *Δt* = *t*^+^ - *t*^-^ = 30 min). Using the abbreviations $c_{t/a}^{\pm }=c_{t/a}(t^{\pm })$ and Equation 2, *K*_*s*_ is given by

(4) $$K_{s}=\frac{m_{t}}{c_{a}^{0}}=\frac{1}{c_{a}^{0}}\cdot \frac{\Delta c_{t}}{\mathrm{\Delta t}}=\frac{1}{\sqrt{c_{a}^{-}\cdot c_{a}^{+}}}\cdot \frac{c_{t}^{+}-c_{t}^{-}}{t^{+}-t^{-}},$$

where $c_{a}^{0}$ was calculated from the exponential connecting the two points $\left(t^{-},c_{a}^{-}\right)$ and $\left(t^{+},c_{a}^{+}\right)$ which yields $c_{a}^{0}=\sqrt{c_{a}^{-}\cdot c_{a}^{+}}$.

For $K_{s}^{\left(c\right)}$ computation according to Equation 3, we fixed *V*_*r*_ to the mean of the Patlak evaluation for all lesions (${\bar{V}}_{r}=0.53$ ml/ml), while *τ*_*a*_ was estimated individually for each study from the exponential connecting $\left(t^{-},c_{a}^{-}\right)$ and $\left(t^{+},c_{a}^{+}\right)$ as

$$\tau_{a}=\frac{\mathrm{\Delta t}}{\ln(c_{a}^{-}/c_{a}^{+})}.$$

Additionally, the retention index was computed as $\text{RI}=\Delta c_{t}/c_{t}^{-}$. Linear regression analysis was performed between *K*_*m*_ and *K*_*s*_, $K_{s}^{\left(c\right)}$, RI, and $c_{t}^{+}$ (the SUV of the lesions in the late image), respectively. Parametric images of *K*_*s*_, $K_{s}^{\left(c\right)}$, and *K*_*m*_ were generated for visual comparison after filtering of the DTP image data with a bilateral filter [16] (spatial filter width 9 mm, intensity filter width 2.5 SUV).

### Influence of image noise

Considering a single voxel and neglecting the (much smaller) statistical error of the ROI-based $c_{a}^{0}$ value, it follows from Equation 4 that the relative statistical error of *K*_*s*_ is equal to that of *Δc*_*t*_ and thus, by Gaussian error propagation,

$$\frac{\sigma_{K_{s}}}{K_{s}}=\frac{\sqrt{{\left[\sigma_{c_{t}^{+}}\right]}^{2}+{\left[\sigma_{c_{t}^{-}}\right]}^{2}}}{c_{t}^{+}-c_{t}^{-}}$$

which decreases with increasing concentration difference *Δc*_*t*_. Taking into account that measurement times of both dual time point measurements might be adjusted in such a way that $\sigma_{c_{t}^{-}}\approx \sigma_{c_{t}^{+}}$, one can get a rough estimate of the error according to

$$\frac{\sigma_{K_{s}}}{K_{s}}\approx \sqrt{2}\cdot \frac{\sigma_{c_{t}^{+}}}{c_{t}^{+}-c_{t}^{-}}=\sqrt{2}\cdot \frac{1}{1-\frac{c_{t}^{-}}{c_{t}^{+}}}\cdot \frac{\sigma_{c_{t}^{+}}}{c_{t}^{+}},$$

where the final ratio represents the relative SUV error of the second dual time point measurement. For *Δt* ≈ 30 min and typical tumor accumulation rates of ≈ 2% to 4% per minute, one can thus estimate that the relative errors of *K*_*s*_ are about 2.5 to 4 times higher than the corresponding SUV errors (the statistical error of $K_{s}^{\left(c\right)}$ is quite similar since the small correction term ${\bar{V}}_{r}/\tau_{a}$ cannot contribute much to the total statistical uncertainty of $K_{s}^{\left(c\right)})$. Although the noise in the parametric maps can thus be expected to be distinctly higher than that in the uptake images, the resulting visual quality is still quite satisfactory for reasonable choices of *Δt*(≳30 min) as will be demonstrated in the following.

## Results

**Figure 2:**
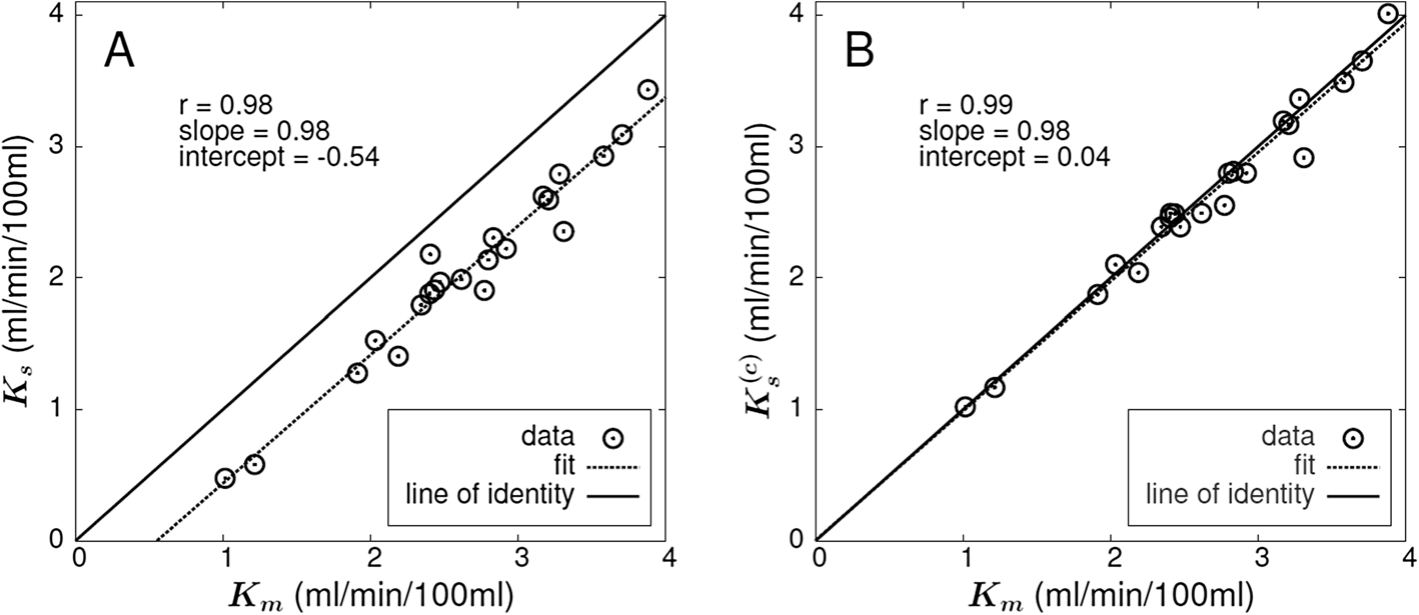
**Correlation between the metabolic rate *K***_***m ***_**and the DTP-derived rate constants. (A) ***K*_*m*_ and *K*_*s*_ and **(B) ***K*_*m*_ and $K_{s}^{\left(c\right)}$. Solid black lines represent the line of identity; dashed lines represent the least squares straight line fits to the data.

The obtained results are summarized in Tables 1 and 2. Figure 2A shows the correlation between Patlak-derived *K*_*m*_ and *K*_*s*_. The solid line is the line of identity, and the dashed line is the linear regression result. The linear correlation is very good, and the fitted slope is identical to one within the given error limits of about 5% (Table 2). The fitted intercept of -0.54 ml/min/100 ml thus represents the experimentally observed average underestimate of the true *K*_*m*_ by *K*_*s*_.

**Table 1:** **Summary of parameters entering the *K***_***s ***_**and **$K_{s}^{\left(c\right)}$**determination**

	**Mean ± SD**	**Range**
*V*_*r *_(ml/ml)	0.53 ± 0.08	0.39 - 0.68
Dynamic *τ*_*a*_ (min)	104 ± 20	79 - 156
DTP *τ*_*a*_ (min)	99 ± 23	81 - 172
$c_{a}^{0}$ (SUV)	3.1 ± 0.7	2.4 - 4.5

‘Dynamic *τ*_*a*_’ denotes the result derived from the full dynamic AIF data with *t* > 20 min. ‘DTP *τ*_*a*_’ was computed solely from the used DTP data. Only the latter was used in further computations.

**Table 2:** **Linear regression results: Pearson correlation coefficient *r * and the obtained regression parameters are shown**

	***r***	**Slope (mean ± SD)**	**Intercept (mean ± SD)**
*K*_*m*_ vs. *K*_*s*_	0.98	0.98 ± 0.05	-0.54 ± 0.13
*K*_*m*_ vs. $K_{s}^{\left(c\right)}$	0.99	0.98 ± 0.04	0.04 ± 0.10
*K*_*m*_ vs. $c_{t}^{+}$	0.60	1.62 ± 0.48	2.81 ± 1.33
*K*_*m*_ vs. RI	0.88	11.38 ± 1.34	5.44 ± 3.79

All correlations were found to be statistically significant with *p* < 0.005. Chosen units are in milliliters per minute per 100 ml for *K*_*m*_, *K*_*s*_, and $K_{s}^{\left(c\right)}$; SUV for $c_{t}^{+}$; and percentage for RI (from which the respective slope and intercept units follow).

Figure 2B presents the correlation between *K*_*m*_ and $K_{s}^{\left(c\right)}=K_{s}+K_{0}$ according to Equation 3. The correction term $K_{0}={\bar{V}}_{r}/\tau_{a}$ was computed using the average *V*_*r*_ derived from the Patlak analysis of all 22 lesions, ${\bar{V}}_{r}=0.53$ ml/ml, and individual (investigation-specific) time constants *τ*_*a*_ derived from the DTP data (the independent determination of *τ*_*a*_ from the full dynamic data in the Patlak time window - performed as a consistency check - yielded essentially the same result (104 ± 20 min (dynamic) vs. 99 ± 23 min (DTP)) but was not used further). As can be seen, the degree of linear correlation is distinctly improved in comparison to Figure 2A. Furthermore, the fitted straight line now essentially coincides with the line of identity. Consequently, the average difference between *K*_*m*_ and $K_{s}^{\left(c\right)}$ amounts to only 1.4 ± 4.1% and exceeds 10% only in a single lesion.

**Figure 3:**
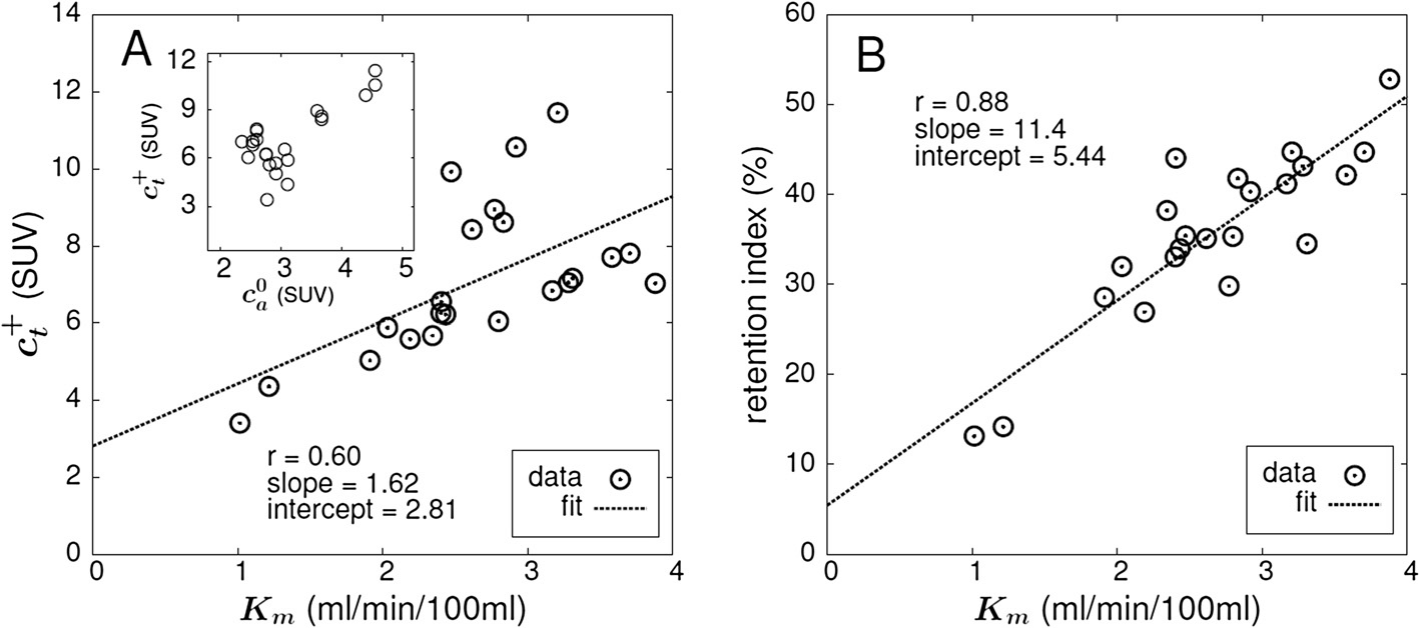
**Correlation between the metabolic rate *K***_***m ***_**and Standard Uptake Value and retention index, respectively.** (A) *K*_*m*_ and $c_{t}^{+}$ (in SUV units) and (B) *K*_*m*_ and retention index (in percent). $c_{t}^{+}$ is the tissue uptake at *t* = 55 min. Dashed lines represent the least squares straight line fits to the data. The inset graph in (A) additionally provides the correlation between $c_{a}^{0}$ and $c_{t}^{+}$, which helps to explain the group of data points above the regression line with SUV > 8 (see the ‘Discussion’ section).

For comparison, Figure 3A,B presents the correlations between *K*_*m*_ and the late SUV uptake $c_{t}^{+}$, and *K*_*m*_ and the retention index RI, respectively. Obviously, the correlation between *K*_*m*_ and $c_{t}^{+}$ is rather poor. The correlation between *K*_*m*_ and the RI is substantially higher but still clearly below the degree of correlation between *K*_*m*_ and *K*_*s*_ or $K_{s}^{\left(c\right)}$. The correlation in Figure 3A is clearly distorted by the group of the six highest observed SUV values which correspond to only moderately high *K*_*m*_ values. This phenomenon might be explained by the exceptionally high $c_{a}^{0}$ values observed in the respective patients (see inset graphic in Figure 3A).

**Figure 4:**
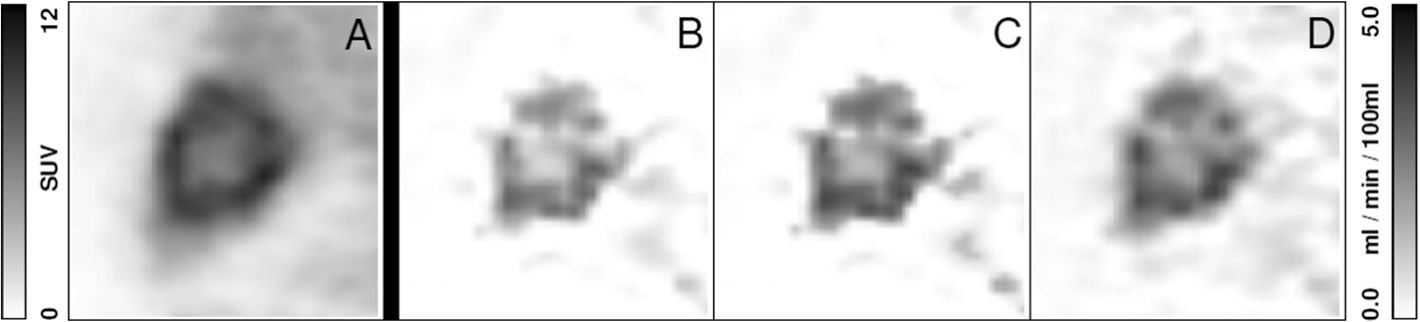
**Liver metastasis of a colorectal carcinoma exhibiting a central necrosis.** (**A**) A representative sagittal slice of uptake and the corresponding parametric images of (**B**) *K*_*s*_, (**C**) $K_{s}^{\left(c\right)}$, and (**D**) *K*_*m*_, respectively, are shown. The means of the rate constants over the lesion are as follows: *K*_*s*_ = 2.23 ml/min/100 ml, $K_{s}^{\left(c\right)}=2.80$ ml/min/100 ml, and *K*_*m*_ = 2.90 ml/min/100 ml, demonstrating a quite satisfactory quantitative agreement between $K_{s}^{\left(c\right)}$ and *K*_*m*_.

Figure 4A provides one example of a lesion uptake image, and the corresponding parametric images of *K*_*s*_, $K_{s}^{\left(c\right)}$, and *K*_*m*_ are shown in Figure 4B,C,D, respectively, all of which are displayed in a common scale. The comparable, enhanced target-to-background contrast of the three parametric images relative to the uptake image is obvious. In agreement with the theoretical expectation and the ROI data in Figure 2, there is good quantitative concordance between *K*_*m*_ and $K_{s}^{\left(c\right)}$, while *K*_*s*_ exhibits a constant negative bias of about *K*_0_ ≈ -0.5 ml/min/100 ml in comparison to *K*_*m*_.

**Figure 5:**
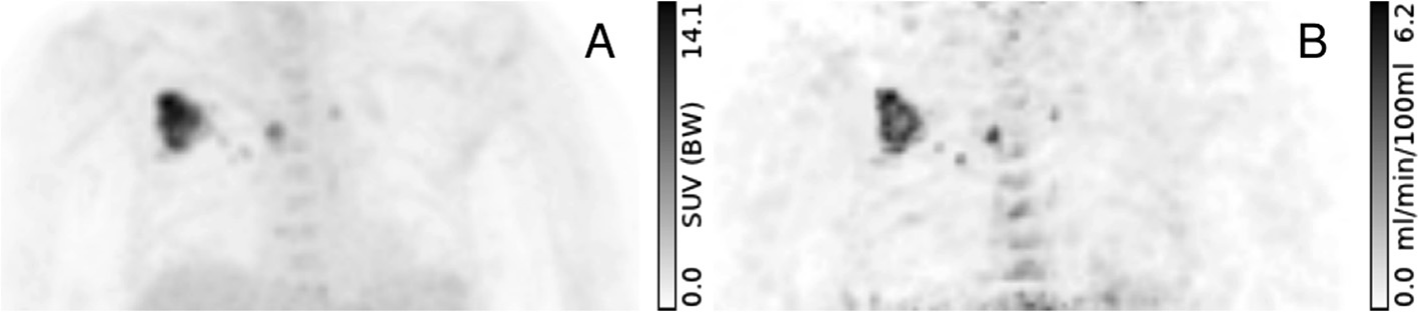
**Comparison of parametric images of SUV and**$K_{s}^{\left(c\right)}$**.(A)** FDG SUV 67 min. p.i. and **(B)** parametric $K_{s}^{\left(c\right)}$ map in a 63-year-old woman. The patient has a bronchial carcinoma of the right lung and lymph node metastases of the right hilar region and the mediastinum (coronal maximum projection). Note the different regional contrast and the used units in both images.

Finally, Figure 5 demonstrates the feasibility of generating parametric $K_{s}^{\left(c\right)}$ maps of reasonable statistical quality for a typical DTP whole-body FDG study.

## Discussion

Our main result is that in the investigated patient group, there is a very pronounced linear correlation *K*_*s*_ = *a* + *b* · *K*_*m*_, where *b* is very nearly equal to one (see Figure 2A). This behavior is in complete agreement with the formalism presented in the Appendix, notably Equation 13: the variations of the (small) term *V*_*r*_ / *τ*_*a*_ should be essentially uncorrelated to *K*_*m*_ so that a high (but slightly “noisy”) linear correlation between *K*_*m*_ and *K*_*s*_ with a slope near one is predicted. Furthermore, according to Equation 13, the modulus of the intercept, *a*=-0.0054 ml/min/ml, should be approximately equal to the average of *V*_*r*_/*τ*_*a*_ in the investigated patient group. This prediction, too, is in complete agreement with the actual values of *V*_*r*_ (determined from Patlak analysis) and *τ*_*a*_, namely ${\bar{V}}_{r}=(0.53\pm 0.08)$ ml/ml and ${\bar{\tau }}_{a}=(99\pm 23)$ min.

The second important finding is the fact that the degree of correlation as well as quantitative agreement between *K*_*m*_ and *K*_*s*_ can be further improved by assuming a reasonable constant value for ${\bar{V}}_{r}$ (since *V*_*r*_ is inaccessible in DTP measurements) and determining individually the rate of decrease, *τ*_*a*_, of the AIF (which can be estimated from the DTP measurement). The corrected *K*_*s*_, $K_{s}^{\left(c\right)}=K_{s}+{\bar{V}}_{r}/\tau_{a}$, exhibits an improved correlation to *K*_*m*_ (due to compensation of the *τ*_*a*_ variability) and also improved quantitative concordance as long as ${\bar{V}}_{r}$ is roughly in accord with the individual true *V*_*r*_. Both phenomena are illustrated clearly in Figure 2B. The distinctly improved correlation (compared to Figure 2A) is achieved by the individual correction of the *τ*_*a*_ influence. The residual deviations from the perfect correlation in Figure 2B are mainly due to the variability of *V*_*r*_. A nearly perfect quantitative agreement with *K*_*m*_ is observed since ${\bar{V}}_{r}$ was set to the mean of the actual *V*_*r*_ values derived from the Patlak analysis. This obviously would not be possible when considering realistic DTP measurements (without a preceding complete dynamic study), and a less-than-perfect quantitative agreement should be expected in this case. Nevertheless, as the comparison of Figure 2A,B suggests, performing the correction with some roughly correct value for ${\bar{V}}_{r}$ will always decrease the bias between *K*_*s*_ and *K*_*m*_.

The rather small variability of tumor *V*_*r*_ observed in the present investigation might seem surprising. However, the square of *k*_2_ / (*k*_2_ + *k*_3_) appearing in Equation 6 will never deviate very much from unity since for FDG, *k*_2_ quite generally is distinctly larger than *k*_3_. The variability of *V*_*r*_ is thus mostly controlled by the first term, *K*_1_ / *k*_2_. Since both *K*_1_ and *k*_2_ are usually identified as being associated with the facilitated diffusion across the cell membrane, it might very well be expected that the ratio *K*_1_ / *k*_2_ is essentially constant, independent of the actual *K*_1_. This might be the underlying reason for the low variability of *V*_*r*_ observed in this study. Whether *V*_*r*_ variability is higher in other tumors remains to be investigated, but we believe this to be unlikely. *V*_*r*_ should never be much larger than about 0.6 to 0.7 ml/ml which appears to be a rough upper bound for the *K*_1_ / *k*_2_ ratio. According to our own data, this is true, e.g., in the human brain (*K*_1_ / *k*_2_ ≈ 0.1 / 0.15 = 0.67 ml/ml) as well as the myocardium (*K*_1_ / *k*_2_ ≈ 0.6 / 1.4 = 0.43 ml/ml). *V*_*r*_ in these organs is rather low (≈ 0.3 ml/ml) due to the large *k*_3_ in both tissues.

**Figure 6:**
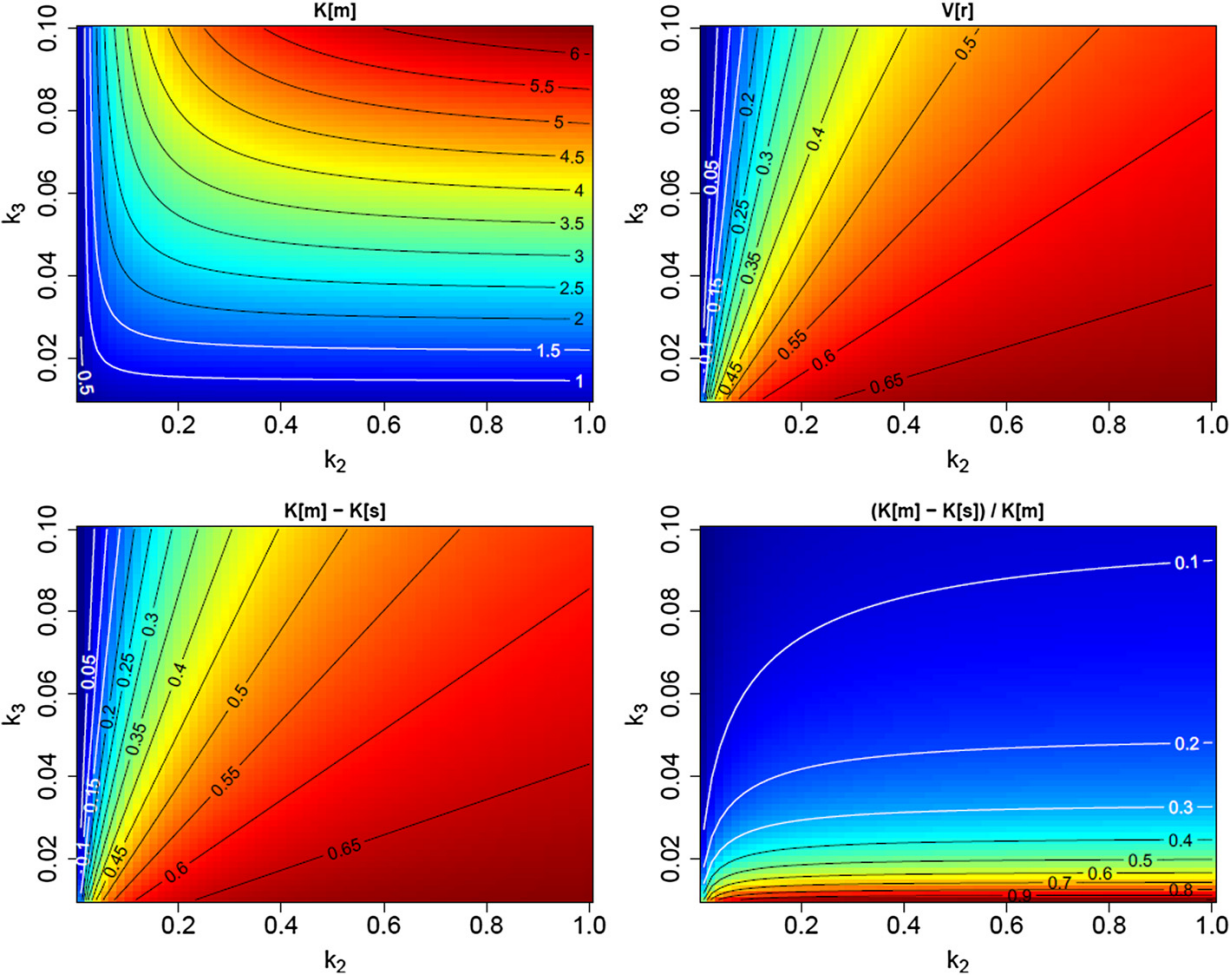
**Visualization of the difference between *K***_***m ***_**and *K***_***s ***_**.** The plots cover a substantial range of the parameters *k*_2_ and *k*_3_, assuming a fixed ratio *K*_1_ / *k*_2_ = 0.7 ml/ml (a rationale for fixing this ratio is given in the discussion above). Top left, *K*_*m*_; top right, *V*_*r*_; bottom left, absolute difference (*K*_*m*_ - *K*_*s*_); and bottom right, fractional difference ((*K*_*m*_ - *K*_*s*_) / *K*_*m*_). Parameters and their respective units: *k*_2_, *k*_3_ (1/min); *V*_*r*_ (ml/ml); and *K*_*m*_, *K*_*s*_ (ml/min/100 ml). Moving along the line, *V*_*r*_ ≈ 0.55 ml/ml between *K*_*m*_ = 1 and 4 ml/min/100 ml corresponds approximately to the experimental data of Figure 2A.

**Figure 7:**
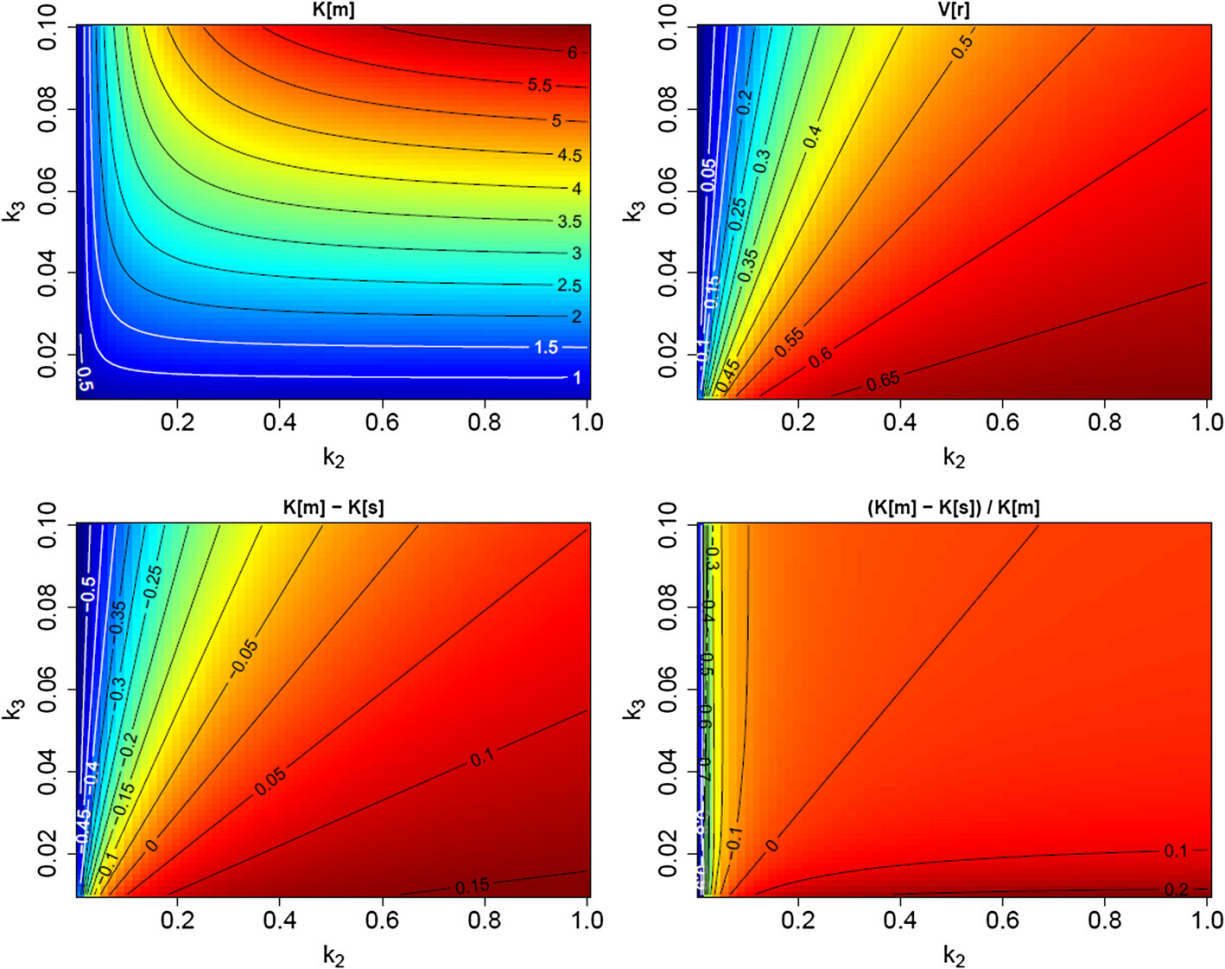
**Visualization of the difference between *K***_***m ***_**and**$K_{s}^{\left(c\right)}$**for *τ***_***a ***_**= 99 min and an assumed distribution volume**${\bar{V}}_{r}=0.53$**ml/ml.** In comparison with Figure 6, the systematic difference relative to the true *K*_*m*_ is mostly removed even when *V*_*r*_ deviates distinctly from the assumed value. Moving along the line, *V*_*r*_ ≈ 0.55 ml/ml between *K*_*m*_ = 1 and 4 ml/min/100ml corresponds approximately to the experimental data of Figure 2B.

We surmise, therefore, that *V*_*r*_ in tumors (and healthy tissue) will never deviate too much from the value of 0.53, ml/ml used for *K*_*s*_ correction in this study. The corrected rate, $K_{s}^{\left(c\right)}$, can then be expected to be a less biased estimate of *K*_*m*_ than *K*_*s*_ over a substantial range of actually realized *V*_*r*_ values between about 0.2 and 0.7 ml/ml (see Figures 6 and 7). Whether $K_{s}^{\left(c\right)}$ does offer any advantages over *K*_*s*_ in terms of clinical relevance remains to be seen, but the improved correlation with *K*_*m*_ seems justification enough to perform the correction.

The very high correlation between $K_{s}^{\left(c\right)}$ (or *K*_*s*_) and *K*_*m*_ is to be compared with the markedly inferior correlation between *K*_*m*_ and late SUV ($c_{t}^{+}$) and retention index RI, respectively (Figure 3). Since all these parameters are ultimately intended as surrogate parameters of *K*_*m*_, the superiority of *K*_*s*_ seems obvious. Since in the present study the retention index is computed from exactly the same DTP tissue data as *K*_*s*_, it is worth to point out that the sole factor responsible for the much better *K*_*s*_(*K*_*m*_) correlation is adequate consideration of the substantial inter-subject $c_{a}^{0}$ variability (see Table 1). Indeed, one could write $K_{s}=\Delta c_{t}/\mathrm{\Delta t}/c_{a}^{0}=\Delta c_{t}/c_{t}^{-}/\mathrm{\Delta t}\cdot c_{t}^{-}/c_{a}^{0}=\text{RI}/\mathrm{\Delta t}\cdot c_{t}^{-}/c_{a}^{0}$, where *Δt* is just a constant in the present context. To some extent, *K*_*s*_ might thus be considered just a more sensible definition of a retention index where the uptake difference *Δc*_*t*_ is normalized to $c_{a}^{0}$ (as well as *Δt*) instead of $c_{t}^{-}$.

The observed very low correlation between late SUV and *K*_*m*_ is caused by the six data points with SUV >8 in Figure 3A. Leaving these six points out increases the correlation coefficient to 0.94 which is in good agreement with published data [17]. Closer inspection revealed exceptionally high $c_{a}^{0}$ values (see inset graphic in Figure 3A) for the affected data points which might have physiological reasons but could also hint at erroneous SUV calibration (for which, however, a retrospective inspection did not find any evidence). In any case, the data demonstrate the high sensitivity of SUV evaluations to variations of the AIF level and incorrect SUV calibration.

The comparison of uptake and parametric images in Figure 4 demonstrates that *K*_*s*_ as well as $K_{s}^{\left(c\right)}$ reproduces the essential features of the Patlak *K*_*m*_ image, notably the increased contrast between metastasis and liver background. Regarding the targeted lesions, the $K_{s}^{\left(c\right)}$ image is, moreover, in good quantitative agreement with the *K*_*m*_ image and could thus serve as a basis for regional quantitative evaluation. We, therefore, believe it is worthwhile to investigate the potential suitability of $K_{s}^{\left(c\right)}$ as a quantitative estimator (and not just a surrogate) of *K*_*m*_ more thoroughly in future studies. Figure 5 demonstrates that reasonable statistical quality of the $K_{s}^{\left(c\right)}$ map can in fact be achieved in whole-body DTP investigations as well.

Compared to more conventional approaches, our approach has several relevant benefits. The most important one in our view is the potential to perform fully quantitative whole-body investigations based on a DTP acquisition. The only additional prerequisite is identification of the aorta or left ventricle in the DTP data. One gains the ability to directly identify regions of elevated irreversible FDG metabolism and to put the established DTP approach on a quantitative basis. A further advantage is the implied correction for the sizable inter-subject variation of the blood tracer concentration (SUV range, 2.4-4.5 in this study). The latter correction alone clearly improves the correlation between the derived parameter (*K*_*s*_) and the targeted one (*K*_*m*_). Another important aspect is elimination of the dependence of SUV uptake and retention index on the time of measurement(s). To the extent that the Patlak model can be considered valid (negligible *k*_4_), the proposed procedure yields a time-independent result, namely a direct estimate of the invariant rate *K*_*m*_ which prospectively should allow definition of improved, objective reference values. A further implication is elimination of any intra-scan time dependence in whole-body/multi-bed studies. Last but not least, the issue of ensuring correct SUV calibration is eliminated since all calibration factors cancel out when performing an image-based determination of both TRF slope and $c_{a}^{0}$. This observation seems especially relevant for multi-center studies.

## Conclusion

We have demonstrated that it is possible to derive a quantitative estimate of *K*_*m*_, the metabolic trapping rate of FDG, solely from a dual time point measurement. We believe this approach to be of potential relevance especially in the context of oncological whole-body investigations where the required AIF information is available in the field of view (aorta or left ventricle). In this case, the approach eliminates most if not all issues of static SUV and conventional dual time point imaging regarding the influence of the chosen scan times relative to the time of injection and the substantial influence of inter-study variability of the AIF. Consequently, the derived parameters *K*_*s*_ and $K_{s}^{\left(c\right)}$ exhibit a much improved stability and much better correlation with the true *K*_*m*_. These properties might prove especially relevant in the context of radiation treatment planning and therapy response control. Whether this is indeed the case has to be investigated in appropriate future studies.

## Appendix

We start with the standard Patlak formula but avoid division by *c*_*a*_(*t*):^a^

(5) $$\begin{array}{c} c_{t}(t)=K_{m}\cdot \int_{0}^{t}c_{a}(s)\text{ds}+V_{r}\cdot c_{a}(t), \end{array}$$

where *K*_*m*_ is the metabolic trapping rate, defined by

$$K_{m}=\frac{K_{1}k_{3}}{k_{2}+k_{3}}$$

and *V*_*r*_ is the apparent volume of distribution defined by

(6) $$V_{r}=\frac{K_{1}k_{2}}{{\left(k_{2}+k_{3}\right)}^{2}}=\frac{K_{1}}{k_{2}}\cdot {\left(\frac{k_{2}}{k_{2}+k_{3}}\right)}^{2}.$$

Equation 5 is valid for times *t* > *T*^∗^ where *T*^∗^ ≈ 20 to 30 min p.i.. Utilization of this equation for *K*_*m*_ determination requires measurements of the TRF only for *t* > *T*^∗^ but measurement of the complete AIF starting at time zero. We now want to eliminate the dependency on measurements prior to *T*^∗^. By taking the time derivative at some time point *t* > *T*^∗^, it follows directly from Equation 5 that

$${ċ}_{t}(t)=K_{m}\cdot c_{a}(t)+V_{r}\cdot {ċ}_{a}(t)$$

or after division by *c*_*a*_(*t*) (suppressing the *t* argument)

(7) $$\frac{{ċ}_{t}}{c_{a}}=K_{m}+V_{r}\cdot \frac{{ċ}_{a}}{c_{a}}.$$

Focusing on some specific time point *t* = *t*_0_, we use the Taylor expansion of *c*_*a*_(*t*) around $t_{0}\;(c_{a}^{\left(n\right)}(t_{0})$: *n*th derivative at *t* = *t*_0_):

(8) $$c_{a}(t)=\overset{\infty }{\underset{n=0}{\sum }}\frac{c_{a}^{\left(n\right)}(t_{0})}{n!}{\left(t-t_{0}\right)}^{n}.$$

Introducing the parameters *τ*_*n*_ defined by ${\left(-\tau_{n}\right)}^{n}=c_{a}^{0}/c_{a}^{\left(n\right)}(t_{0})$, Equation 8 can be rewritten as

(9) $$\begin{array}{ll} \;c_{a}(t) & =c_{a}^{0}\cdot \overset{\infty }{\underset{n=0}{\sum }}\frac{{\left(-1\right)}^{n}}{n!}{\left(\frac{t-t_{0}}{\tau_{n}}\right)}^{n}\\ & =c_{a}^{0}\cdot \left(1-\frac{t-t_{0}}{\tau_{1}}+\frac{1}{2!}{\left(\frac{t-t_{0}}{\tau_{2}}\right)}^{2}-\ldots \right), \end{array}$$

where *τ*_0_ is always equal to one. The parameters *τ*_*n* > 0_ are constructed in such a way that for a mono-exponential decrease of *c*_*a*_(*t*) near *t*_0_, we obtain *τ*_*n* > 0_ = *τ*_*a*_, where *τ*_*a*_ is the time constant of the exponential. Actually, it is known that starting rather early after bolus injection (*t* > 20 min), *c*_*a*_(*t*) can be reasonably well described by a slow mono-exponential decrease with a time constant *τ*_*a*_ ≈ 100 min (in the present study, we found an average value of *τ*_*a*_ = 99 min, while a value of *τ*_*a*_ = 80 min was reported in [18]).

Inserting the Taylor expansion from Equation 9 into Equation 7, we get (${ċ}_{t}^{0}={ċ}_{t}(t_{0})$)

(10) $$\frac{{ċ}_{t}^{0}}{c_{a}^{0}}=K_{m}-\frac{V_{r}}{\tau_{1}}.$$

In order to derive *K*_*m*_ from this equation, we need to reliably estimate ${ċ}_{t}^{0}/c_{a}^{0}$ as well as to have knowledge of 1 / *τ*_1_ (the fractional rate of decrease of the AIF at *t* = *t*_0_). Obviously, direct determination of the time derivative ${ċ}_{t}^{0}$ at *t* = *t*_0_ is not feasible in real (noisy) data. On the other hand, it is not clear whether the average slope over a necessarily rather large neighborhood (required for reasons of limited time resolution and count rate statistics) is an acceptable approximation of ${ċ}_{t}^{0}$ (since the slope changes over time). For investigation of this question, we compute from Equation 5 the difference $c_{t}^{+}-c_{t}^{-}=c_{t}\left(t_{0}+\frac{\mathrm{\Delta t}}{2}\right)-c_{t}\left(t_{0}-\frac{\mathrm{\Delta t}}{2}\right)$ for two time points lying symmetrically around *t*_0_ at a finite (possibly large) distance *Δt*

(11) $$\;\Delta c_{t}(\mathrm{\Delta t})=c_{t}^{+}-c_{t}^{-}=K_{m}\cdot \int_{t_{0}-\frac{\mathrm{\Delta t}}{2}}^{t_{0}+\frac{\mathrm{\Delta t}}{2}}c_{a}(s)\text{ds}+V_{r}\cdot \left(c_{a}^{+}-c_{a}^{-}\right)$$

with $c_{a}^{\pm }=c_{a}\left(t_{0}\pm \frac{\mathrm{\Delta t}}{2}\right)$.

Replacing all occurrences of *c*_*a*_(*t*) in Equation 11 by the Taylor series in Equation 9 (neglecting fourth and higher order terms) and executing the integration separately for each term of the series yield after some straightforward but lengthy calculations the following equation:

(12) $$\begin{array}{ll} \;\Delta c_{t}= & \left(K_{m}\left[1+\frac{1}{24}{\left(\frac{\mathrm{\Delta t}}{\tau_{2}}\right)}^{2}\right]\right)\\ & \left(-\frac{V_{r}}{\tau_{1}}\left[1+\frac{1}{24}\frac{\tau_{1}}{\tau_{3}}{\left(\frac{\mathrm{\Delta t}}{\tau_{3}}\right)}^{2}\right]\right)c_{a}^{0}\cdot \mathrm{\Delta t} \end{array}$$

The detailed derivation of Equation 12 is presented in an additional file (see Additional file 1). The factors in square brackets deviate only minimally from one up to even quite large values of *Δt*. For the sake of simplicity, we will demonstrate this only for the well-established approximately mono-exponential behavior of *c*_*a*_(*t*) at later times but emphasize that the conclusions remain the same when using other reasonable parametrizations of the observed shape of the AIF at later times (e.g., by an inverse power law).

As already pointed out, for a mono-exponential decrease of *c*_*a*_(*t*), all *τ*_*n* > 0_ coincide with the time constant *τ*_*a*_ of the exponential. Consider, then, choosing *Δt* = 60 min in Equation 12. Since *τ*_*a*_ ≈ 100 min, we have for both square brackets 1 + 1 / 24 · 0.6^2^ = 1.015. It is, therefore, permissible to replace both square brackets by one. This yields

$$\Delta c_{t}\approx \left[K_{m}-\frac{V_{r}}{\tau_{a}}\right]c_{a}^{0}\cdot \mathrm{\Delta t.}$$

Thus, *Δc*_*t*_ is to a very good approximation proportional to *Δt*. *Δt* can become quite large, e.g., *Δt* = 1 h, as long as the lower bound $t_{0}-\frac{\mathrm{\Delta t}}{2}$ remains larger than *T*^∗^. Defining the secant slope *m*_*t*_ between $c_{t}^{-}$ and $c_{t}^{+}$

$$m_{t}=\frac{\Delta c_{t}}{\mathrm{\Delta t}}$$

and introducing the rate constant *K*_*s*_

$$K_{s}=\frac{m_{t}}{c_{a}^{0}}$$

for the ratio of the secant slope and the blood concentration at *t*_0_, we get

(13) $$K_{s}=\frac{m_{t}}{c_{a}^{0}}=K_{m}-\frac{V_{r}}{\tau_{a}}$$

or

(14) $$K_{m}=K_{s}+\frac{V_{r}}{\tau_{a}}.$$

Comparison of Equation 13 with Equation 10 yields the important result

$$m_{t}={ċ}_{t}^{0}.$$

In other words, the secant slope is to a very good approximation equal to the instantaneous slope at *t*_0_ and thus can be used instead. This in turn implies that the average slope of the TRF (derivable, e.g., by a least squares fit of a straight line in the considered time window), too, is very nearly identical to *m*_*t*_. Note that these conclusions are valid even if ${ċ}_{t}(t)$ varies considerably over the considered time interval (see Figure 1). Formally, this result is identical to stating that a second-order Taylor expansion of *c*_*t*_(*t*) around *t*_0_ turns out to be sufficiently accurate within *t*_0_ ± *Δt* / 2.

The quantitative relation between *K*_*s*_ and *K*_*m*_ is investigated in Figure 6. For this figure, we computed *K*_*m*_ and *V*_*r*_ over a range of sensible choices for the transport constants *K*_1_, *k*_2_, and *k*_3_. The resulting *K*_*m*_ and *V*_*r*_ (top row of Figure 6) are used to compute *K*_*s*_ from Equation 13 for a realistic value of *τ*_*a*_ (we chose *τ*_*a*_ = 99 min). The bottom row in Figure 6 compares the true *K*_*m*_ to *K*_*s*_.

As can be seen (bottom right), the fractional deviation of *K*_*s*_ from *K*_*m*_ becomes large only when *k*_3_ is very small (i.e., when there is virtually no trapping). Overall *K*_*s*_ is a negatively biased estimator of *K*_*m*_, but an approximate correction of the bias is possible considering the following.

According to Equation 14, the *V*_*r*_ and *K*_*m*_ - *K*_*s*_ maps in Figure 6 differ only by a constant factor *τ*_*a*_ (and a conversion factor of 100 due to the chosen units of ml/min/100 ml for *K*_*m*_ and *K*_*s*_). Moreover, *V*_*r*_ does vary only modestly in comparison to the individual rate constants and to *K*_*m*_ (except when *k*_3_ becomes distinctly larger than *k*_2_, but this is not observed in real data). Therefore, *K*_*m*_ - *K*_*s*_ does not vary much across the relevant part of the *k*_2_ / *k*_3_ plane. We, therefore, hypothesize that the difference *K*_*m*_ - *K*_*s*_ can be actually treated to be approximately constant. Consequently, we propose to estimate *K*_*m*_ using only late time measurements of *c*_*a*_(*t*) and *c*_*t*_(*t*) as follows: 

1. Determine the secant TRF slope *m*_*t*_ in the time interval $t^{\pm }=t_{0}\pm \frac{\mathrm{\Delta t}}{2}$ from a dual time point measurement of *c*_*t*_(*t*) starting at sufficiently late times after injection, typically *t* > (20-30) min.

2. Estimate $c_{a}^{0}=c_{a}(t_{0})$ and *τ*_*a*_ from the exponential connecting the two time points *t*^ - ^, *t*^+^.

3. Compute $K_{s}=m_{t}/c_{a}^{0}$.

4. Compute a correction term $K_{0}={\bar{V}}_{r}/\tau_{a}$ using the individually determined *τ*_*a*_ and a fixed value ${\bar{V}}_{r}$ for the distribution volume. In the absence of any specific information regarding *V*_*r*_ in the investigated tumor entity, we propose to use the average *V*_*r*_ determined in this study, i.e., ${\bar{V}}_{r}=0.53$ ml/ml.

5. Finally, compute the corrected *K*_*s*_, i.e.,

(15) $$K_{s}^{\left(c\right)}=K_{s}+K_{0}=K_{s}+{\bar{V}}_{r}/\tau_{a}$$

as a quantitative estimate of the true *K*_*m*_.

According to Equations 14 and 15, $K_{s}^{\left(c\right)}$ is equal to *K*_*m*_ if $V_{r}={\bar{V}}_{r}$ (irrespective of the values of *K*_1_ - *k*_3_ yielding this *V*_*r*_ value). Therefore, $K_{s}^{\left(c\right)}$ remains a very good approximation of *K*_*m*_ as long as *V*_*r*_ does not deviate too much from the assumed value. This behavior is illustrated in Figure 7.

## Endnote

^a^For completeness, we mention that in the presence of sizable fractional blood volume (*fbv*), the substitutions $K_{m}\to K_{m}^{*}=(1-\text{fbv})K_{m}$ and $V_{r}\to V_{r}^{*}=(1-\text{fbv})V_{r}+\text{fbv}=V_{r}+\text{fbv}(1-V_{r})$ would have to be performed in Equation 5 where the ‘asterisked’ quantities would be the experimentally accessible ones.

## Competing interests

The authors declare that they have no competing interests.

## Authors’ contributions

JVDH derived the theoretical background, performed part of the data analysis, and is the main author of the manuscript. FH performed part of the data analysis and wrote part of the manuscript. LO and GS contributed to the derivation of the theoretical background. JL and BBB performed the PET measurements. JS and JK provided intellectual input and reviewed the manuscript. All authors read and approved the final manuscript.

## Supplementary Material

Additional file 1 **Derivation of Equation** 12**.** A pdf file showing the complete derivation of Equation 12 using Taylor expansion. Click here for file
