# Supplementary material for: Dual time point based quantification of metabolic uptake rates in 18F-FDG PET
Source: EJNMMI Res. 2013 Mar 13;3:16. doi: 10.1186/2191-219X-3-16 (PMC3717002; doi:10.1186/2191-219X-3-16)
Supplement: Additional file 1 — Derivation of Equation 12. A pdf file showing the complete derivation of Equation 12 using Taylor expansion. [file 2191-219X-3-16-S1.pdf]

Derivation of Eq. 12 in

"Dual time point based quantification of metabolic uptake rates in ^{18}F-FDG PET"

J. van den Hoff et. al.

Starting point is Eq. 11 in the publication

$$\Delta c_t(\Delta t) = c_t^+ - c_t^- = K_m \cdot \int_{t_0-\frac{\Delta t}{2}}^{t_0+\frac{\Delta t}{2}} c_a(s)ds + V_r \cdot (c_a^+ - c_a^-) \tag{1}$$

with $c_a^\pm = c_a(t_0 \pm \frac{\Delta t}{2})$. Using the Taylor expansion of c_a(t)

$$c_a(t) = c_a^0 \cdot \sum_{n=0}^{\infty} \frac{(-1)^n}{n!} \left(\frac{t-t_0}{\tau_n}\right)^n$$

the integral in Eq. 1 can be calculated:

$$\begin{aligned}\int_{t_0-\frac{\Delta t}{2}}^{t_0+\frac{\Delta t}{2}} c_a(s)ds &= c_a^0 \cdot \sum_{n=0}^{\infty} \frac{(-1)^n}{n!} \int_{t_0-\frac{\Delta t}{2}}^{t_0+\frac{\Delta t}{2}} \left(\frac{s-t_0}{\tau_n}\right)^n \\ &= c_a^0 \cdot \sum_{n=0}^{\infty} \frac{(-1)^n}{n!} \frac{\tau_n}{n+1} \left(\frac{s-t_0}{\tau_n}\right)^{n+1} \Bigg|_{t_0-\frac{\Delta t}{2}}^{t_0+\frac{\Delta t}{2}} \\ &= c_a^0 \cdot \sum_{n=0}^{\infty} \frac{(-1)^n}{(n+1)!} \cdot \tau_n \cdot \left[\left(\frac{\Delta t}{2\tau_n}\right)^{n+1} - \left(-\frac{\Delta t}{2\tau_n}\right)^{n+1}\right] .\end{aligned}$$

All terms with even powers of $\frac{\Delta t}{2\tau_n}$ vanish (i.e. when n is odd) resulting in

$$\begin{aligned}\int_{t_0-\frac{\Delta t}{2}}^{t_0+\frac{\Delta t}{2}} c_a(s)ds &= c_a^0 \cdot \left[\Delta t + \frac{\tau_2}{24}\left(\frac{\Delta t}{\tau_2}\right)^3 + ...\right] \\ &= c_a^0 \cdot \Delta t \cdot \left[1 + \frac{1}{24}\left(\frac{\Delta t}{\tau_2}\right)^2 + ...\right] .\end{aligned} \tag{2}$$

where the first two non-vanishing terms of the sum are explicitely given and higher order terms are indicated by the ellipsis.

The difference term $(c_a^+ - c_a^-)$ is computed accordingly:

$$\begin{aligned}c_a^+ - c_a^- &= c_a^0 \cdot \sum_{n=0}^{\infty} \frac{(-1)^n}{n!} \left[\left(\frac{\Delta t}{2\tau_n}\right)^n - \left(-\frac{\Delta t}{2\tau_n}\right)^n\right] \\ &= -c_a^0 \cdot \left[\frac{\Delta t}{\tau_1} + \frac{1}{24}\left(\frac{\Delta t}{\tau_3}\right)^3 + ...\right] \\ &= -c_a^0 \cdot \Delta t \cdot \frac{1}{\tau_1}\left[1 + \frac{1}{24}\frac{\tau_1}{\tau_3}\left(\frac{\Delta t}{\tau_3}\right)^2 + ...\right]\end{aligned} \tag{3}$$

where again all terms with even powers of $\frac{\Delta t}{2\tau_n}$ vanish (i.e. when n is even). Inserting (2) and (3) in (1) and neglecting fourth and higher order terms leads to Eq. 12 in the publication:

$$\Delta c_t = \left(K_m \left[1 + \frac{1}{24}\left(\frac{\Delta t}{\tau_2}\right)^2\right] - \frac{V_r}{\tau_1}\left[1 + \frac{1}{24}\frac{\tau_1}{\tau_3}\left(\frac{\Delta t}{\tau_3}\right)^2\right]\right) c_a^0 \cdot \Delta t \,.$$
